# Crystal structures of vortioxetine and its methanol monosolvate

**DOI:** 10.1107/S2056989015012256

**Published:** 2015-07-04

**Authors:** Xin-Bo Zhou, Jian-Ming Gu, Meng-ying Sun, Xiu-Rong Hu, Su-Xiang Wu

**Affiliations:** a College of Pharmaceutical Science, Zhejiang Chinese Medical University, Hangzhou, Zhejiang 310053, People’s Republic of China; b Center of Analysis and Measurement, Zhejiang University, Hangzhou, Zhejiang 310028, People’s Republic of China; c Chemistry Department, Zhejiang University, Hangzhou, Zhejiang 310028, People’s Republic of China

**Keywords:** crystal structure, vortioxetine, major depressive disorder, hydrogen bonding

## Abstract

Vortioxetine, C_18_H_22_N_2_S, (1), systematic name 1-{2-[(2,4-di­methyl­phen­yl)sulfan­yl]phen­yl}piperazine, a new drug used to treat patients with major depressive disorder, has been crystallized as the free base and its methanol monosolvate, C_18_H_22_N_2_S·CH_3_OH, (2). In both structures, the vortioxetine mol­ecules have similar conformations: in (1), the dihedral angle between the aromatic rings is 80.04 (16)° and in (2) it is 84.94 (13)°. The C—S—C bond angle in (1) is 102.76 (14)° and the corresponding angle in (2) is 103.41 (11)°. The piperazine ring adopts a chair conformation with the exocyclic N—C bond in a pseudo-equatorial orientation in both structures. No directional inter­actions beyond normal van der Waals contacts could be identified in the crystal of (1), whereas in (2), the vortioxetine and methanol mol­ecules are linked by N—H⋯O and O—H⋯N hydrogen bonds, generating [001] chains.

## Abstract 2

Vortioxetine, a new drug used to treat patients with major depressive disorder, has been crystallized as the free base and its methanol monosolvate. In both structures, the vortioxetine mol­ecules have similar conformations.

## Chemical context   

Major depressive disorder (MDD) is a disabling mental illness responsible for almost 66 million disability-adjusted life-years globally (Bidzan *et al.*, 2012 ▸). The medications most often prescribed for depression include the selective serotonin reuptake inhibitors (SSRIs) and the serotonin norepinephrine reuptake inhibitors (SNRIs). As several neurotransmitter pathways may be involved in MDD, anti­depressants possessing two or more complementary modes of action (*i.e.* multi-modal) have been a focus of MDD therapy for some time (Richelson, 2013 ▸). One such anti­depressant is vortioxetine.
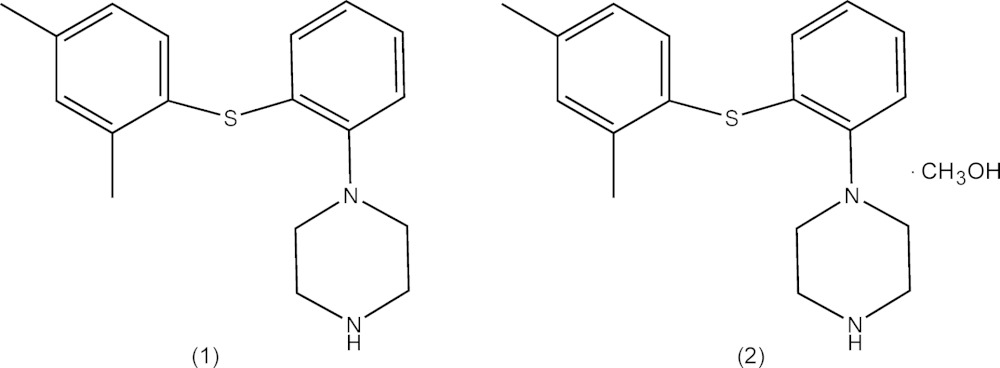


Vortioxetine is an investigational multi-modal anti­depressant that is believed to work through a combination of two pharmacological modes of action: serotonin (5-HT) reuptake inhibition and 5-HT receptor activity (du Jardin *et al.*, 2014 ▸; Hussar *et al.*, 2014 ▸). In 2013, vortioxetine hydro­bromide was approved by the US Food and Drug Administration (FDA) for the once-daily treatment of adults with MDD in the USA (Gibb & Deeks, 2014 ▸. The patent of Benny *et al.* (2007 ▸) discloses crystalline vortioxetine base and a variety of crystalline vortioxetine salts, comprising polymorphs of vortioxetine hydro­bromide as well as a hemihydrate and an ethyl acetate solvate thereof, and crystalline vortioxetine hydro­chloride and a monohydrate thereof. Crystalline vortioxetine mesylate, meso­hydrogentartrate, hydrogenmaleate and hydrogen sulfate are also disclosed. However, there are few reports on the single-crystal X-ray structure of vortioxetine base and its salts. As part of our ongoing structural studies of pharmaceutical compounds, the crystal structures of vortioxetine free base (1), and its methanol solvate (2), have been determined and reported here.

## Structural commentary   

**Figure 1:**
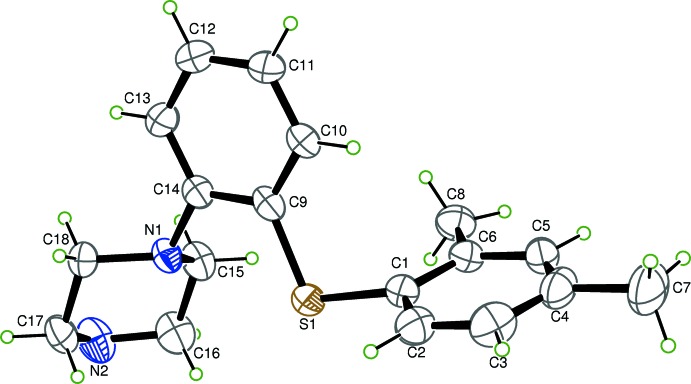
The mol­ecular structure of compound (1), showing 50% probability displacement ellipsoids.

The asymmetric unit of (1) consists of one vortioxetine mol­ecule and that of compound (2) consists of one vortioxetine mol­ecule and one methanol mol­ecule. Views of the asymmetric units of (1) and (2), with atom labelling, are presented in Figs. 1 ▸ and 2 ▸, respectively. In both structures, the two benzene rings bridged by the S atom, are almost perpendicular to one another. The dihedral angles between the planes of these benzene rings is 80.04 (16)° in compound (1) and 84.94 (13)° in compound(2). The S atom is nearly coplanar with the benzene rings as indicated by C1—S1—C9—C14 torsion angles of 176.0 (2) for (1) and −176.04 (18)° for (2). The piperazine ring of both structures adopts a chair conformation with the exocyclic N1—C14 bond in a pseudo equatorial orientation. Atoms N1 and N2 deviate from the best fit plane through the remaining four C atoms by 0.683 (1) and 0.637 (1) Å in (1) and by 0.698 (1) and −0.562 Å in (2).

## Supra­molecular features   

**Figure 3:**
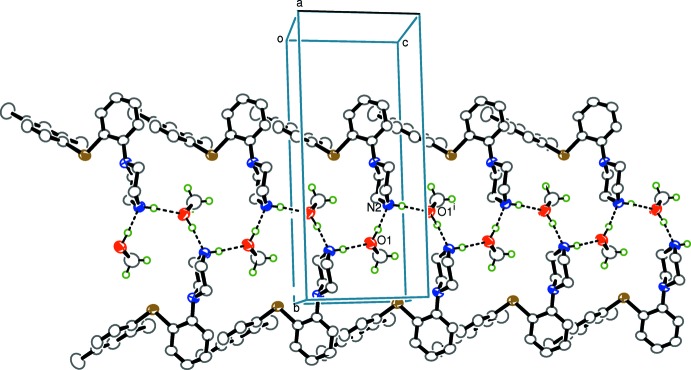
Part of the crystal packing of compound (2), viewed along the *a* axis. Hydrogen bonds are shown as dashed lines. H atoms not involved in hydrogen bonding have been omitted for clarity.

There are no hydrogen bonds or π–π stacking inter­actions linking the mol­ecules in (1), while in (2) the presence of the additional methanol solvent mol­ecule results in the formation of zigzag chains mediated by alternating O1—H1⋯N2 and N2—H2*A*⋯O1^i^ [symmetry code: (i) *x*, −*y* + 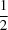, *z* + 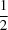] hydrogen bonds propagating along the *c-*axis direction (Table 1 ▸). a packing diagram for (2) is shown in Fig. 3 ▸.

## Synthesis and crystallization   

Vortioxetine was supplied by Zhejiang Jingxin Pharmaceutical Co., Ltd. Crystals of (1) and (2) suitable for X-ray diffraction were recrystallized by slow evaporation from aceto­nitrile and methanol–water solutions, respectively, at room temperature over a few days.

## Refinement   

Crystal data, data collection and structure refinement details are summarized in Table 2 ▸. All H atoms were placed in idealized positions and refined as riding, with C—H = 0.93–0.97, N—H = 0.86 and O—H = 0.82 Å and *U*_iso_(H) = 1.2*U*_eq_ or 1.5*U*_eq_(carrier atom).

## Supplementary Material

Crystal structure: contains datablock(s) 1, 2, global. DOI: 10.1107/S2056989015012256/hb7395sup1.cif

Structure factors: contains datablock(s) 1, 2. DOI: 10.1107/S2056989015012256/hb73951sup3.hkl

Structure factors: contains datablock(s) 2. DOI: 10.1107/S2056989015012256/hb73952sup4.hkl

Supporting information file. DOI: 10.1107/S2056989015012256/hb73951sup4.cml

Supporting information file. DOI: 10.1107/S2056989015012256/hb73952sup5.cml

CCDC references: 1408949, 1408948

Additional supporting information:  crystallographic information; 3D view; checkCIF report

## Figures and Tables

**Figure 2:**
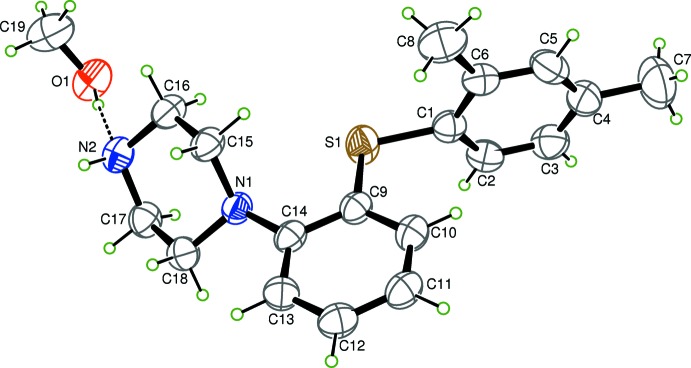
The mol­ecular structure of compound (2), showing 50% probability displacement ellipsoids.

**Table 1:** Hydrogen-bond geometry (Å, °) for (2)

*D*—H⋯*A*	*D*—H	H⋯*A*	*D*⋯*A*	*D*—H⋯*A*
N2—H2*A*⋯O1^i^	0.86	2.15	2.930 (3)	151
O1—H1⋯N2	0.82	1.93	2.744 (3)	171

Symmetry code: (i) 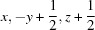.

**Table 2:** Experimental details

	(1)	(2)
Crystal data		
Chemical formula	C_18_H_22_N_2_S	C_18_H_22_N_2_S·CH_4_O
*M*_r_	298.44	330.48
Crystal system, space group	Triclinic, *P* 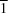	Monoclinic, *P*2_1_/*c*
Temperature (K)	296	296
*a*, *b*, *c* (Å)	7.6160 (4), 8.3267 (5), 13.9011 (7)	13.2100 (7), 18.1500 (9), 8.1746 (4)
α, β, γ (°)	84.999 (2), 77.631 (1), 74.347 (2)	90, 104.378 (2), 90
*V* (Å^3^)	828.75 (8)	1898.57 (17)
*Z*	2	4
Radiation type	Mo *K*α	Mo *K*α
μ (mm^−1^)	0.19	0.18
Crystal size (mm)	0.48 × 0.38 × 0.16	0.38 × 0.33 × 0.28

Data collection		
Diffractometer	Rigaku R-AXIS RAPID/ZJUG	Rigaku R-AXIS RAPID/ZJUG
Absorption correction	Multi-scan (*ABSCOR*: Higashi, 1995 ▸)	Multi-scan (*ABSCOR*: Higashi, 1995 ▸)
*T*_min_, *T*_max_	0.904, 0.970	0.928, 0.952
No. of measured, independent and observed [*I* > 2σ(*I*)] reflections	8178, 3756, 2072	18365, 4331, 2468
*R*_int_	0.053	0.054
(sin θ/λ)_max_ (Å^−1^)	0.649	0.648

Refinement		
*R*[*F* ^2^ > 2σ(*F* ^2^)], *wR*(*F* ^2^), *S*	0.056, 0.185, 1.00	0.052, 0.156, 1.00
No. of reflections	3756	4331
No. of parameters	193	213
H-atom treatment	H-atom parameters constrained	H-atom parameters constrained
Δρ_max_, Δρ_min_ (e Å^−3^)	0.40, −0.37	0.25, −0.25

Computer programs: *PROCESS-AUTO* and *CrystalStructure* (Rigaku, 2007 ▸), *SHELXS97* and *SHELXL97* (Sheldrick, 2008 ▸) and *ORTEP-3 for Windows* and *WinGX* (Farrugia, 2012 ▸).

## supplementary crystallographic information

### (1) 1-{2-[(2,4-Dimethylphenyl)sulfanyl]phenyl}piperazine Crystal data

**Table:**

C_18_H_22_N_2_S	*Z* = 2
*M**_r_* = 298.44	*F*(000) = 320
Triclinic, *P*1	*D*_x_ = 1.196 Mg m^−^^3^
Hall symbol: -P 1	Mo *K*α radiation, λ = 0.71073 Å
*a* = 7.6160 (4) Å	Cell parameters from 5189 reflections
*b* = 8.3267 (5) Å	θ = 3.0–27.4°
*c* = 13.9011 (7) Å	µ = 0.19 mm^−^^1^
α = 84.999 (2)°	*T* = 296 K
β = 77.631 (1)°	Chunk, colorless
γ = 74.347 (2)°	0.48 × 0.38 × 0.16 mm
*V* = 828.75 (8) Å^3^	

### (1) 1-{2-[(2,4-Dimethylphenyl)sulfanyl]phenyl}piperazine Data collection

**Table:**

Rigaku R-AXIS RAPID/ZJUG diffractometer	3756 independent reflections
Radiation source: rotating anode	2072 reflections with *I* > 2σ(*I*)
Graphite monochromator	*R*_int_ = 0.053
Detector resolution: 10.00 pixels mm^-1^	θ_max_ = 27.5°, θ_min_ = 3.0°
ω scans	*h* = −9→9
Absorption correction: multi-scan (ABSCOR: Higashi, 1995)	*k* = −10→10
*T*_min_ = 0.904, *T*_max_ = 0.970	*l* = −18→17
8178 measured reflections	

### (1) 1-{2-[(2,4-Dimethylphenyl)sulfanyl]phenyl}piperazine Refinement

**Table:**

Refinement on *F*^2^	Secondary atom site location: difference Fourier map
Least-squares matrix: full	Hydrogen site location: inferred from neighbouring sites
*R*[*F*^2^ > 2σ(*F*^2^)] = 0.056	H-atom parameters constrained
*wR*(*F*^2^) = 0.185	*w* = 1/[σ^2^(*F*_o_^2^) + (0.0622*P*)^2^ + 0.7569*P*] where *P* = (*F*_o_^2^ + 2*F*_c_^2^)/3
*S* = 1.00	(Δ/σ)_max_ < 0.001
3756 reflections	Δρ_max_ = 0.40 e Å^−^^3^
193 parameters	Δρ_min_ = −0.37 e Å^−^^3^
0 restraints	Extinction correction: SHELXL97 (Sheldrick, 2008), Fc^*^=kFc[1+0.001xFc^2^λ^3^/sin(2θ)]^-1/4^
Primary atom site location: structure-invariant direct methods	Extinction coefficient: 0.072 (7)

### (1) 1-{2-[(2,4-Dimethylphenyl)sulfanyl]phenyl}piperazine Special details

**Table:**

Geometry. All e.s.d.'s (except the e.s.d. in the dihedral angle between two l.s. planes) are estimated using the full covariance matrix. The cell e.s.d.'s are taken into account individually in the estimation of e.s.d.'s in distances, angles and torsion angles; correlations between e.s.d.'s in cell parameters are only used when they are defined by crystal symmetry. An approximate (isotropic) treatment of cell e.s.d.'s is used for estimating e.s.d.'s involving l.s. planes.
Refinement. Refinement of *F*^2^ against ALL reflections. The weighted *R*-factor *wR* and goodness of fit *S* are based on *F*^2^, conventional *R*-factors *R* are based on *F*, with *F* set to zero for negative *F*^2^. The threshold expression of *F*^2^ > σ(*F*^2^) is used only for calculating *R*-factors(gt) *etc*. and is not relevant to the choice of reflections for refinement. *R*-factors based on *F*^2^ are statistically about twice as large as those based on *F*, and *R*- factors based on ALL data will be even larger.

### (1) 1-{2-[(2,4-Dimethylphenyl)sulfanyl]phenyl}piperazine Fractional atomic coordinates and isotropic or equivalent isotropic displacement parameters (Å^2^)

**Table:**

	*x*	*y*	*z*	*U*_iso_*/*U*_eq_	
C1	0.6391 (4)	0.3576 (4)	0.1355 (2)	0.0428 (7)	
C2	0.6226 (5)	0.1944 (5)	0.1467 (3)	0.0549 (8)	
H2	0.5698	0.1559	0.2079	0.066*	
C3	0.6828 (5)	0.0891 (5)	0.0688 (3)	0.0641 (10)	
H3	0.6711	−0.0198	0.0781	0.077*	
C4	0.7608 (5)	0.1436 (5)	−0.0235 (3)	0.0575 (9)	
C5	0.7753 (4)	0.3054 (5)	−0.0349 (2)	0.0520 (8)	
H5	0.8265	0.3428	−0.0969	0.062*	
C6	0.7165 (4)	0.4163 (4)	0.0425 (2)	0.0446 (7)	
C7	0.8231 (6)	0.0284 (6)	−0.1104 (3)	0.0894 (14)	
H7A	0.8791	0.0843	−0.1673	0.134*	
H7B	0.7174	−0.0007	−0.1241	0.134*	
H7C	0.9121	−0.0710	−0.0948	0.134*	
C8	0.7350 (5)	0.5916 (4)	0.0254 (3)	0.0573 (9)	
H8A	0.8268	0.6061	0.0592	0.086*	
H8B	0.6175	0.6680	0.0498	0.086*	
H8C	0.7727	0.6130	−0.0440	0.086*	
C9	0.7492 (4)	0.5017 (4)	0.2771 (2)	0.0429 (7)	
C10	0.9282 (4)	0.4087 (4)	0.2382 (2)	0.0478 (8)	
H10	0.9473	0.3388	0.1862	0.057*	
C11	1.0770 (4)	0.4193 (4)	0.2761 (2)	0.0526 (8)	
H11	1.1958	0.3563	0.2496	0.063*	
C12	1.0515 (4)	0.5228 (5)	0.3533 (2)	0.0568 (9)	
H12	1.1525	0.5293	0.3789	0.068*	
C13	0.8738 (4)	0.6169 (4)	0.3921 (2)	0.0523 (8)	
H13	0.8565	0.6869	0.4438	0.063*	
C14	0.7213 (4)	0.6081 (4)	0.3550 (2)	0.0420 (7)	
C15	0.4781 (5)	0.8573 (4)	0.3382 (2)	0.0542 (8)	
H15A	0.5081	0.8376	0.2681	0.065*	
H15B	0.5450	0.9350	0.3504	0.065*	
C16	0.2704 (5)	0.9311 (5)	0.3705 (3)	0.0643 (10)	
H16A	0.2338	1.0370	0.3356	0.077*	
H16B	0.2040	0.8566	0.3535	0.077*	
C17	0.2816 (5)	0.8026 (5)	0.5307 (2)	0.0561 (9)	
H17A	0.2164	0.7227	0.5196	0.067*	
H17B	0.2516	0.8238	0.6006	0.067*	
C18	0.4901 (4)	0.7287 (4)	0.4994 (2)	0.0485 (8)	
H18A	0.5568	0.8049	0.5140	0.058*	
H18B	0.5278	0.6242	0.5354	0.058*	
N1	0.5348 (3)	0.7000 (3)	0.39293 (16)	0.0425 (6)	
N2	0.2190 (4)	0.9568 (4)	0.4764 (2)	0.0692 (9)	
H2A	0.1597	1.0506	0.5030	0.083*	
S1	0.54672 (10)	0.48897 (12)	0.23790 (6)	0.0524 (3)	

### (1) 1-{2-[(2,4-Dimethylphenyl)sulfanyl]phenyl}piperazine Atomic displacement parameters (Å^2^)

**Table:**

	*U*^11^	*U*^22^	*U*^33^	*U*^12^	*U*^13^	*U*^23^
C1	0.0376 (15)	0.0479 (18)	0.0434 (15)	−0.0111 (13)	−0.0076 (12)	−0.0052 (13)
C2	0.0553 (19)	0.057 (2)	0.0552 (19)	−0.0189 (16)	−0.0121 (16)	−0.0012 (16)
C3	0.069 (2)	0.048 (2)	0.079 (3)	−0.0141 (17)	−0.022 (2)	−0.0033 (18)
C4	0.0539 (19)	0.058 (2)	0.061 (2)	−0.0015 (16)	−0.0194 (17)	−0.0219 (17)
C5	0.0440 (17)	0.067 (2)	0.0425 (16)	−0.0091 (15)	−0.0087 (14)	−0.0079 (15)
C6	0.0372 (15)	0.0502 (19)	0.0458 (16)	−0.0103 (13)	−0.0077 (13)	−0.0024 (13)
C7	0.088 (3)	0.095 (3)	0.086 (3)	−0.005 (3)	−0.023 (2)	−0.048 (3)
C8	0.055 (2)	0.055 (2)	0.064 (2)	−0.0212 (16)	−0.0089 (16)	0.0014 (17)
C9	0.0402 (15)	0.0448 (18)	0.0375 (14)	−0.0075 (13)	0.0005 (12)	0.0006 (12)
C10	0.0390 (15)	0.056 (2)	0.0437 (16)	−0.0090 (14)	0.0006 (13)	−0.0094 (14)
C11	0.0346 (15)	0.066 (2)	0.0540 (18)	−0.0071 (14)	−0.0076 (14)	−0.0083 (16)
C12	0.0426 (17)	0.074 (3)	0.058 (2)	−0.0168 (16)	−0.0161 (15)	−0.0066 (17)
C13	0.0475 (18)	0.065 (2)	0.0488 (17)	−0.0172 (15)	−0.0110 (15)	−0.0141 (15)
C14	0.0410 (15)	0.0452 (18)	0.0378 (14)	−0.0113 (13)	−0.0035 (12)	−0.0012 (12)
C15	0.059 (2)	0.048 (2)	0.0475 (17)	−0.0077 (15)	−0.0032 (15)	0.0013 (14)
C16	0.064 (2)	0.051 (2)	0.065 (2)	0.0022 (17)	−0.0102 (18)	0.0045 (17)
C17	0.0546 (19)	0.061 (2)	0.0459 (17)	−0.0119 (16)	0.0054 (15)	−0.0107 (15)
C18	0.0516 (18)	0.057 (2)	0.0376 (15)	−0.0142 (15)	−0.0066 (13)	−0.0097 (14)
N1	0.0423 (13)	0.0423 (15)	0.0367 (12)	−0.0054 (11)	−0.0013 (10)	−0.0033 (10)
N2	0.071 (2)	0.0517 (19)	0.0656 (18)	0.0063 (15)	0.0048 (16)	−0.0124 (15)
S1	0.0349 (4)	0.0732 (6)	0.0475 (5)	−0.0108 (4)	−0.0023 (3)	−0.0186 (4)

### (1) 1-{2-[(2,4-Dimethylphenyl)sulfanyl]phenyl}piperazine Geometric parameters (Å, º)

**Table:**

C1—C2	1.391 (5)	C11—C12	1.384 (5)
C1—C6	1.406 (4)	C11—H11	0.9300
C1—S1	1.773 (3)	C12—C13	1.388 (4)
C2—C3	1.373 (5)	C12—H12	0.9300
C2—H2	0.9300	C13—C14	1.389 (4)
C3—C4	1.386 (5)	C13—H13	0.9300
C3—H3	0.9300	C14—N1	1.430 (4)
C4—C5	1.374 (5)	C15—N1	1.464 (4)
C4—C7	1.522 (5)	C15—C16	1.517 (5)
C5—C6	1.398 (4)	C15—H15A	0.9700
C5—H5	0.9300	C15—H15B	0.9700
C6—C8	1.497 (5)	C16—N2	1.459 (4)
C7—H7A	0.9600	C16—H16A	0.9700
C7—H7B	0.9600	C16—H16B	0.9700
C7—H7C	0.9600	C17—N2	1.449 (4)
C8—H8A	0.9600	C17—C18	1.521 (4)
C8—H8B	0.9600	C17—H17A	0.9700
C8—H8C	0.9600	C17—H17B	0.9700
C9—C10	1.392 (4)	C18—N1	1.472 (4)
C9—C14	1.403 (4)	C18—H18A	0.9700
C9—S1	1.773 (3)	C18—H18B	0.9700
C10—C11	1.373 (4)	N2—H2A	0.8600
C10—H10	0.9300		
			
C2—C1—C6	119.3 (3)	C11—C12—H12	120.3
C2—C1—S1	118.4 (2)	C13—C12—H12	120.3
C6—C1—S1	122.2 (2)	C14—C13—C12	121.1 (3)
C3—C2—C1	121.2 (3)	C14—C13—H13	119.5
C3—C2—H2	119.4	C12—C13—H13	119.5
C1—C2—H2	119.4	C13—C14—C9	118.9 (3)
C2—C3—C4	120.6 (4)	C13—C14—N1	123.5 (3)
C2—C3—H3	119.7	C9—C14—N1	117.5 (3)
C4—C3—H3	119.7	N1—C15—C16	109.5 (3)
C5—C4—C3	118.3 (3)	N1—C15—H15A	109.8
C5—C4—C7	121.0 (4)	C16—C15—H15A	109.8
C3—C4—C7	120.7 (4)	N1—C15—H15B	109.8
C4—C5—C6	122.9 (3)	C16—C15—H15B	109.8
C4—C5—H5	118.6	H15A—C15—H15B	108.2
C6—C5—H5	118.6	N2—C16—C15	111.3 (3)
C5—C6—C1	117.7 (3)	N2—C16—H16A	109.4
C5—C6—C8	120.4 (3)	C15—C16—H16A	109.4
C1—C6—C8	121.9 (3)	N2—C16—H16B	109.4
C4—C7—H7A	109.5	C15—C16—H16B	109.4
C4—C7—H7B	109.5	H16A—C16—H16B	108.0
H7A—C7—H7B	109.5	N2—C17—C18	111.5 (3)
C4—C7—H7C	109.5	N2—C17—H17A	109.3
H7A—C7—H7C	109.5	C18—C17—H17A	109.3
H7B—C7—H7C	109.5	N2—C17—H17B	109.3
C6—C8—H8A	109.5	C18—C17—H17B	109.3
C6—C8—H8B	109.5	H17A—C17—H17B	108.0
H8A—C8—H8B	109.5	N1—C18—C17	108.9 (3)
C6—C8—H8C	109.5	N1—C18—H18A	109.9
H8A—C8—H8C	109.5	C17—C18—H18A	109.9
H8B—C8—H8C	109.5	N1—C18—H18B	109.9
C10—C9—C14	119.6 (3)	C17—C18—H18B	109.9
C10—C9—S1	124.1 (2)	H18A—C18—H18B	108.3
C14—C9—S1	116.3 (2)	C14—N1—C15	112.9 (2)
C11—C10—C9	120.5 (3)	C14—N1—C18	115.5 (2)
C11—C10—H10	119.7	C15—N1—C18	110.2 (2)
C9—C10—H10	119.7	C17—N2—C16	110.8 (3)
C10—C11—C12	120.6 (3)	C17—N2—H2A	124.6
C10—C11—H11	119.7	C16—N2—H2A	124.6
C12—C11—H11	119.7	C9—S1—C1	102.76 (14)
C11—C12—C13	119.3 (3)		
			
C6—C1—C2—C3	0.9 (5)	C10—C9—C14—C13	−0.6 (4)
S1—C1—C2—C3	176.7 (3)	S1—C9—C14—C13	176.8 (2)
C1—C2—C3—C4	−0.4 (5)	C10—C9—C14—N1	−179.5 (3)
C2—C3—C4—C5	−0.3 (5)	S1—C9—C14—N1	−2.1 (3)
C2—C3—C4—C7	−178.4 (3)	N1—C15—C16—N2	57.3 (4)
C3—C4—C5—C6	0.6 (5)	N2—C17—C18—N1	−57.9 (4)
C7—C4—C5—C6	178.7 (3)	C13—C14—N1—C15	95.2 (4)
C4—C5—C6—C1	−0.1 (5)	C9—C14—N1—C15	−85.9 (3)
C4—C5—C6—C8	−179.7 (3)	C13—C14—N1—C18	−32.8 (4)
C2—C1—C6—C5	−0.6 (4)	C9—C14—N1—C18	146.1 (3)
S1—C1—C6—C5	−176.2 (2)	C16—C15—N1—C14	169.7 (3)
C2—C1—C6—C8	178.9 (3)	C16—C15—N1—C18	−59.5 (4)
S1—C1—C6—C8	3.3 (4)	C17—C18—N1—C14	−171.1 (3)
C14—C9—C10—C11	0.6 (5)	C17—C18—N1—C15	59.5 (3)
S1—C9—C10—C11	−176.6 (2)	C18—C17—N2—C16	56.1 (4)
C9—C10—C11—C12	−0.1 (5)	C15—C16—N2—C17	−55.7 (4)
C10—C11—C12—C13	−0.3 (5)	C10—C9—S1—C1	−6.7 (3)
C11—C12—C13—C14	0.3 (5)	C14—C9—S1—C1	176.0 (2)
C12—C13—C14—C9	0.1 (5)	C2—C1—S1—C9	106.2 (3)
C12—C13—C14—N1	179.0 (3)	C6—C1—S1—C9	−78.2 (3)

### (2) 1-{2-[(2,4-Dimethylphenyl)sulfanyl]phenyl}piperazine methanol monosolvate Crystal data

**Table:**

C_18_H_22_N_2_S·CH_4_O	*F*(000) = 712
*M**_r_* = 330.48	*D*_x_ = 1.156 Mg m^−^^3^
Monoclinic, *P*2_1_/*c*	Mo *K*α radiation, λ = 0.71073 Å
Hall symbol: -P 2ybc	Cell parameters from 10380 reflections
*a* = 13.2100 (7) Å	θ = 3.2–27.4°
*b* = 18.1500 (9) Å	µ = 0.18 mm^−^^1^
*c* = 8.1746 (4) Å	*T* = 296 K
β = 104.378 (2)°	Chunk, colorless
*V* = 1898.57 (17) Å^3^	0.38 × 0.33 × 0.28 mm
*Z* = 4	

### (2) 1-{2-[(2,4-Dimethylphenyl)sulfanyl]phenyl}piperazine methanol monosolvate Data collection

**Table:**

Rigaku R-AXIS RAPID/ZJUG diffractometer	4331 independent reflections
Radiation source: rotating anode	2468 reflections with *I* > 2σ(*I*)
Graphite monochromator	*R*_int_ = 0.054
Detector resolution: 10.00 pixels mm^-1^	θ_max_ = 27.4°, θ_min_ = 3.2°
ω scans	*h* = −16→17
Absorption correction: multi-scan (ABSCOR: Higashi, 1995)	*k* = −23→23
*T*_min_ = 0.928, *T*_max_ = 0.952	*l* = −10→10
18365 measured reflections	

### (2) 1-{2-[(2,4-Dimethylphenyl)sulfanyl]phenyl}piperazine methanol monosolvate Refinement

**Table:**

Refinement on *F*^2^	Secondary atom site location: difference Fourier map
Least-squares matrix: full	Hydrogen site location: inferred from neighbouring sites
*R*[*F*^2^ > 2σ(*F*^2^)] = 0.052	H-atom parameters constrained
*wR*(*F*^2^) = 0.156	*w* = 1/[σ^2^(*F*_o_^2^) + (0.0598*P*)^2^ + 0.7465*P*] where *P* = (*F*_o_^2^ + 2*F*_c_^2^)/3
*S* = 1.00	(Δ/σ)_max_ = 0.001
4331 reflections	Δρ_max_ = 0.25 e Å^−^^3^
213 parameters	Δρ_min_ = −0.25 e Å^−^^3^
0 restraints	Extinction correction: SHELXL97 (Sheldrick, 2008), Fc^*^=kFc[1+0.001xFc^2^λ^3^/sin(2θ)]^-1/4^
Primary atom site location: structure-invariant direct methods	Extinction coefficient: 0.032 (3)

### (2) 1-{2-[(2,4-Dimethylphenyl)sulfanyl]phenyl}piperazine methanol monosolvate Special details

**Table:**

Geometry. All e.s.d.'s (except the e.s.d. in the dihedral angle between two l.s. planes) are estimated using the full covariance matrix. The cell e.s.d.'s are taken into account individually in the estimation of e.s.d.'s in distances, angles and torsion angles; correlations between e.s.d.'s in cell parameters are only used when they are defined by crystal symmetry. An approximate (isotropic) treatment of cell e.s.d.'s is used for estimating e.s.d.'s involving l.s. planes.
Refinement. Refinement of *F*^2^ against ALL reflections. The weighted *R*-factor *wR* and goodness of fit *S* are based on *F*^2^, conventional *R*-factors *R* are based on *F*, with *F* set to zero for negative *F*^2^. The threshold expression of *F*^2^ > σ(*F*^2^) is used only for calculating *R*-factors(gt) *etc*. and is not relevant to the choice of reflections for refinement. *R*-factors based on *F*^2^ are statistically about twice as large as those based on *F*, and *R*- factors based on ALL data will be even larger.

### (2) 1-{2-[(2,4-Dimethylphenyl)sulfanyl]phenyl}piperazine methanol monosolvate Fractional atomic coordinates and isotropic or equivalent isotropic displacement parameters (Å^2^)

**Table:**

	*x*	*y*	*z*	*U*_iso_*/*U*_eq_	
C16	0.7959 (2)	0.35981 (14)	0.6308 (3)	0.0631 (7)	
H16A	0.7446	0.3322	0.6726	0.076*	
H16B	0.7810	0.3521	0.5097	0.076*	
C15	0.78325 (19)	0.44056 (13)	0.6641 (3)	0.0590 (6)	
H15A	0.7148	0.4573	0.6018	0.071*	
H15B	0.7892	0.4484	0.7835	0.071*	
C18	0.96814 (19)	0.45883 (14)	0.7082 (3)	0.0604 (6)	
H18A	0.9744	0.4667	0.8277	0.073*	
H18B	1.0219	0.4875	0.6754	0.073*	
C17	0.9822 (2)	0.37770 (14)	0.6748 (3)	0.0628 (7)	
H17A	0.9841	0.3717	0.5577	0.075*	
H17B	1.0490	0.3615	0.7446	0.075*	
C14	0.84554 (18)	0.55978 (12)	0.5953 (3)	0.0500 (5)	
C13	0.89399 (19)	0.61073 (14)	0.7155 (3)	0.0579 (6)	
H13	0.9419	0.5948	0.8126	0.069*	
C12	0.8721 (2)	0.68494 (14)	0.6929 (3)	0.0644 (7)	
H12	0.9049	0.7186	0.7747	0.077*	
C11	0.8017 (2)	0.70901 (14)	0.5493 (3)	0.0645 (7)	
H11	0.7865	0.7590	0.5348	0.077*	
C10	0.7535 (2)	0.65945 (13)	0.4267 (3)	0.0600 (6)	
H10	0.7056	0.6761	0.3302	0.072*	
C9	0.77610 (18)	0.58456 (12)	0.4466 (3)	0.0520 (6)	
C1	0.6514 (2)	0.56877 (13)	0.1194 (3)	0.0560 (6)	
C6	0.5482 (2)	0.58898 (14)	0.1102 (3)	0.0612 (6)	
C5	0.4947 (2)	0.62711 (16)	−0.0328 (4)	0.0738 (8)	
H5	0.4261	0.6413	−0.0401	0.089*	
C4	0.5387 (2)	0.64500 (16)	−0.1649 (3)	0.0723 (8)	
C3	0.6398 (2)	0.62274 (17)	−0.1533 (3)	0.0743 (8)	
H3	0.6708	0.6328	−0.2413	0.089*	
C2	0.6953 (2)	0.58576 (15)	−0.0131 (3)	0.0650 (7)	
H2	0.7638	0.5718	−0.0070	0.078*	
C8	0.4960 (3)	0.5711 (2)	0.2494 (4)	0.0946 (10)	
H8A	0.4280	0.5935	0.2252	0.142*	
H8B	0.5376	0.5897	0.3546	0.142*	
H8C	0.4890	0.5186	0.2571	0.142*	
C7	0.4764 (3)	0.6864 (2)	−0.3175 (5)	0.1267 (15)	
H7A	0.5232	0.7121	−0.3704	0.190*	
H7B	0.4311	0.7212	−0.2825	0.190*	
H7C	0.4351	0.6523	−0.3962	0.190*	
C19	0.8246 (3)	0.1517 (2)	0.6374 (5)	0.1043 (11)	
H19A	0.8628	0.1474	0.7534	0.156*	
H19B	0.8153	0.1036	0.5866	0.156*	
H19C	0.7576	0.1734	0.6317	0.156*	
N1	0.86499 (14)	0.48236 (10)	0.6108 (2)	0.0520 (5)	
N2	0.89994 (17)	0.33031 (11)	0.7085 (2)	0.0601 (5)	
H2A	0.9160	0.3296	0.8171	0.090*	
O1	0.87917 (19)	0.19532 (11)	0.5525 (2)	0.0848 (6)	
H1	0.8925	0.2349	0.6017	0.127*	
S1	0.72717 (6)	0.51680 (4)	0.29023 (9)	0.0699 (3)	

### (2) 1-{2-[(2,4-Dimethylphenyl)sulfanyl]phenyl}piperazine methanol monosolvate Atomic displacement parameters (Å^2^)

**Table:**

	*U*^11^	*U*^22^	*U*^33^	*U*^12^	*U*^13^	*U*^23^
C16	0.0652 (16)	0.0537 (14)	0.0744 (16)	−0.0033 (12)	0.0246 (13)	0.0061 (12)
C15	0.0560 (15)	0.0559 (15)	0.0708 (15)	0.0007 (11)	0.0263 (12)	0.0091 (12)
C18	0.0550 (15)	0.0564 (15)	0.0679 (15)	0.0030 (12)	0.0117 (12)	0.0056 (12)
C17	0.0605 (15)	0.0587 (16)	0.0700 (15)	0.0089 (12)	0.0179 (12)	0.0051 (12)
C14	0.0514 (13)	0.0455 (13)	0.0583 (13)	0.0004 (10)	0.0232 (10)	0.0031 (10)
C13	0.0568 (15)	0.0582 (15)	0.0588 (14)	−0.0015 (12)	0.0149 (11)	−0.0027 (11)
C12	0.0692 (17)	0.0522 (15)	0.0726 (16)	−0.0061 (13)	0.0193 (13)	−0.0104 (12)
C11	0.0738 (17)	0.0441 (13)	0.0795 (17)	0.0015 (12)	0.0265 (14)	−0.0004 (12)
C10	0.0660 (16)	0.0479 (14)	0.0657 (15)	0.0032 (12)	0.0158 (12)	0.0040 (11)
C9	0.0538 (14)	0.0469 (13)	0.0585 (13)	0.0014 (11)	0.0197 (11)	0.0017 (10)
C1	0.0587 (15)	0.0494 (13)	0.0603 (14)	−0.0020 (11)	0.0154 (11)	−0.0061 (10)
C6	0.0576 (15)	0.0588 (15)	0.0684 (15)	−0.0071 (12)	0.0183 (12)	−0.0078 (12)
C5	0.0520 (15)	0.0746 (19)	0.087 (2)	0.0034 (13)	0.0023 (14)	−0.0083 (15)
C4	0.076 (2)	0.0682 (18)	0.0647 (16)	−0.0061 (15)	0.0024 (14)	0.0004 (13)
C3	0.079 (2)	0.084 (2)	0.0612 (16)	−0.0154 (16)	0.0194 (14)	−0.0003 (14)
C2	0.0552 (15)	0.0723 (18)	0.0686 (16)	−0.0039 (13)	0.0173 (12)	−0.0070 (13)
C8	0.089 (2)	0.106 (3)	0.102 (2)	−0.012 (2)	0.0492 (19)	−0.0035 (19)
C7	0.138 (4)	0.125 (3)	0.094 (3)	0.014 (3)	−0.014 (2)	0.026 (2)
C19	0.111 (3)	0.101 (3)	0.113 (3)	−0.031 (2)	0.049 (2)	−0.009 (2)
N1	0.0503 (11)	0.0459 (11)	0.0609 (11)	0.0022 (9)	0.0158 (9)	0.0081 (8)
N2	0.0708 (14)	0.0516 (12)	0.0599 (12)	0.0050 (10)	0.0201 (10)	0.0058 (9)
O1	0.1255 (18)	0.0678 (13)	0.0686 (12)	−0.0129 (12)	0.0384 (12)	−0.0116 (9)
S1	0.0871 (5)	0.0482 (4)	0.0669 (4)	0.0070 (3)	0.0050 (3)	−0.0044 (3)

### (2) 1-{2-[(2,4-Dimethylphenyl)sulfanyl]phenyl}piperazine methanol monosolvate Geometric parameters (Å, º)

**Table:**

C16—N2	1.465 (3)	C9—S1	1.776 (2)
C16—C15	1.507 (4)	C1—C2	1.384 (3)
C16—H16A	0.9700	C1—C6	1.395 (4)
C16—H16B	0.9700	C1—S1	1.774 (2)
C15—N1	1.471 (3)	C6—C5	1.391 (4)
C15—H15A	0.9700	C6—C8	1.506 (4)
C15—H15B	0.9700	C5—C4	1.386 (4)
C18—N1	1.461 (3)	C5—H5	0.9300
C18—C17	1.517 (3)	C4—C3	1.376 (4)
C18—H18A	0.9700	C4—C7	1.513 (4)
C18—H18B	0.9700	C3—C2	1.373 (4)
C17—N2	1.464 (3)	C3—H3	0.9300
C17—H17A	0.9700	C2—H2	0.9300
C17—H17B	0.9700	C8—H8A	0.9600
C14—C13	1.385 (3)	C8—H8B	0.9600
C14—C9	1.403 (3)	C8—H8C	0.9600
C14—N1	1.428 (3)	C7—H7A	0.9600
C13—C12	1.380 (3)	C7—H7B	0.9600
C13—H13	0.9300	C7—H7C	0.9600
C12—C11	1.375 (4)	C19—O1	1.370 (4)
C12—H12	0.9300	C19—H19A	0.9600
C11—C10	1.379 (3)	C19—H19B	0.9600
C11—H11	0.9300	C19—H19C	0.9600
C10—C9	1.393 (3)	N2—H2A	0.8598
C10—H10	0.9300	O1—H1	0.8200
			
N2—C16—C15	114.3 (2)	C2—C1—S1	118.1 (2)
N2—C16—H16A	108.7	C6—C1—S1	122.3 (2)
C15—C16—H16A	108.7	C5—C6—C1	117.5 (2)
N2—C16—H16B	108.7	C5—C6—C8	120.7 (3)
C15—C16—H16B	108.7	C1—C6—C8	121.8 (3)
H16A—C16—H16B	107.6	C4—C5—C6	123.2 (3)
N1—C15—C16	109.0 (2)	C4—C5—H5	118.4
N1—C15—H15A	109.9	C6—C5—H5	118.4
C16—C15—H15A	109.9	C3—C4—C5	117.7 (3)
N1—C15—H15B	109.9	C3—C4—C7	121.5 (3)
C16—C15—H15B	109.9	C5—C4—C7	120.8 (3)
H15A—C15—H15B	108.3	C2—C3—C4	120.6 (3)
N1—C18—C17	109.0 (2)	C2—C3—H3	119.7
N1—C18—H18A	109.9	C4—C3—H3	119.7
C17—C18—H18A	109.9	C3—C2—C1	121.5 (3)
N1—C18—H18B	109.9	C3—C2—H2	119.3
C17—C18—H18B	109.9	C1—C2—H2	119.3
H18A—C18—H18B	108.3	C6—C8—H8A	109.5
N2—C17—C18	114.0 (2)	C6—C8—H8B	109.5
N2—C17—H17A	108.8	H8A—C8—H8B	109.5
C18—C17—H17A	108.8	C6—C8—H8C	109.5
N2—C17—H17B	108.8	H8A—C8—H8C	109.5
C18—C17—H17B	108.8	H8B—C8—H8C	109.5
H17A—C17—H17B	107.7	C4—C7—H7A	109.5
C13—C14—C9	119.2 (2)	C4—C7—H7B	109.5
C13—C14—N1	123.6 (2)	H7A—C7—H7B	109.5
C9—C14—N1	117.2 (2)	C4—C7—H7C	109.5
C12—C13—C14	120.8 (2)	H7A—C7—H7C	109.5
C12—C13—H13	119.6	H7B—C7—H7C	109.5
C14—C13—H13	119.6	O1—C19—H19A	109.5
C11—C12—C13	120.0 (2)	O1—C19—H19B	109.5
C11—C12—H12	120.0	H19A—C19—H19B	109.5
C13—C12—H12	120.0	O1—C19—H19C	109.5
C12—C11—C10	120.4 (2)	H19A—C19—H19C	109.5
C12—C11—H11	119.8	H19B—C19—H19C	109.5
C10—C11—H11	119.8	C14—N1—C18	117.31 (19)
C11—C10—C9	120.3 (2)	C14—N1—C15	113.85 (18)
C11—C10—H10	119.9	C18—N1—C15	109.97 (18)
C9—C10—H10	119.9	C17—N2—C16	111.36 (19)
C10—C9—C14	119.3 (2)	C17—N2—H2A	101.4
C10—C9—S1	124.22 (18)	C16—N2—H2A	114.7
C14—C9—S1	116.39 (17)	C19—O1—H1	109.5
C2—C1—C6	119.5 (2)	C1—S1—C9	103.41 (11)
			
N2—C16—C15—N1	54.9 (3)	C6—C5—C4—C7	179.9 (3)
N1—C18—C17—N2	−55.2 (3)	C5—C4—C3—C2	−1.6 (4)
C9—C14—C13—C12	2.2 (4)	C7—C4—C3—C2	179.4 (3)
N1—C14—C13—C12	179.8 (2)	C4—C3—C2—C1	0.9 (4)
C14—C13—C12—C11	−0.3 (4)	C6—C1—C2—C3	0.6 (4)
C13—C12—C11—C10	−0.6 (4)	S1—C1—C2—C3	176.9 (2)
C12—C11—C10—C9	−0.4 (4)	C13—C14—N1—C18	−28.7 (3)
C11—C10—C9—C14	2.3 (4)	C9—C14—N1—C18	149.0 (2)
C11—C10—C9—S1	−174.67 (19)	C13—C14—N1—C15	101.7 (3)
C13—C14—C9—C10	−3.1 (3)	C9—C14—N1—C15	−80.6 (3)
N1—C14—C9—C10	179.1 (2)	C17—C18—N1—C14	−166.5 (2)
C13—C14—C9—S1	174.04 (18)	C17—C18—N1—C15	61.3 (3)
N1—C14—C9—S1	−3.7 (3)	C16—C15—N1—C14	164.7 (2)
C2—C1—C6—C5	−1.3 (4)	C16—C15—N1—C18	−61.3 (3)
S1—C1—C6—C5	−177.42 (19)	C18—C17—N2—C16	48.1 (3)
C2—C1—C6—C8	179.2 (3)	C15—C16—N2—C17	−48.2 (3)
S1—C1—C6—C8	3.0 (4)	C2—C1—S1—C9	100.2 (2)
C1—C6—C5—C4	0.6 (4)	C6—C1—S1—C9	−83.6 (2)
C8—C6—C5—C4	−179.9 (3)	C10—C9—S1—C1	1.0 (2)
C6—C5—C4—C3	0.9 (4)	C14—C9—S1—C1	−176.04 (18)

### (2) 1-{2-[(2,4-Dimethylphenyl)sulfanyl]phenyl}piperazine methanol monosolvate Hydrogen-bond geometry (Å, º)

**Table:**

*D*—H···*A*	*D*—H	H···*A*	*D*···*A*	*D*—H···*A*
N2—H2*A*···O1^i^	0.86	2.15	2.930 (3)	151
O1—H1···N2	0.82	1.93	2.744 (3)	171

Symmetry code: (i) *x*, −*y*+1/2, *z*+1/2.
